# Adipose-Derived Mesenchymal Stem Cells Attenuate Immune Reactions Against Pig Decellularized Bronchi Engrafted into Rat Tracheal Defects

**DOI:** 10.1080/15476278.2023.2212582

**Published:** 2023-05-15

**Authors:** Makoto Hisanaga, Tomoshi Tsuchiya, Hironosuke Watanabe, Koichiro Shimoyama, Mayumi Iwatake, Yukinori Tanoue, Keizaburo Maruyama, Hiroshi Yukawa, Kazuhide Sato, Yoshimi Kato, Keitaro Matsumoto, Takuro Miyazaki, Ryoichiro Doi, Koichi Tomoshige, Takeshi Nagayasu

**Affiliations:** a Department of Surgery, Division of Surgical Oncology, Nagasaki University Graduate School of Biomedical Sciences, Nagasaki, Japan; b Department of Thoracic Surgery, Faculty of Medicine, Academic Assembly, University of Toyama, Toyama, Japan; c Institute of Nano-Life-Systems, Institutes of Innovation for Future Society, Nagoya University, Nagoya, Japan; d Institute of Quantum Life Science, Quantum Life and Medical Siceince Directorate, National Institure for Quantum Science and Technology, Chiba, Japan; e Medical-Engineering Hybrid Professional Development Center, Nagasaki University Graduate School of Biomedical Sciences, Nagasaki, Japan

**Keywords:** Adipose-derived mesenchymal stem cells, biocompatible materials, decellularization, immunosuppression, tissue engineering, xenotransplantation

## Abstract

Decellularized scaffolds are promising biomaterials for tissue and organ reconstruction; however, strategies to effectively suppress the host immune responses toward these implants, particularly those without chemical crosslinking, remain warranted. Administration of mesenchymal stem cells is effective against immune-mediated inflammatory disorders. Herein, we investigated the effect of isogeneic abdominal adipose-derived mesenchymal stem/stromal cells (ADMSCs) on xenogeneic biomaterial-induced immunoreactions. Peripheral bronchi from pigs, decellularized using a detergent enzymatic method, were engrafted onto tracheal defects of Brown Norway (BN) rats. BN rats were implanted with native pig bronchi (Xenograft group), decellularized pig bronchi (Decellularized Xenograft), or Decellularized Xenograft and ADMSCs (Decellularized Xenograft+ADMSC group). In the latter group, ADMSCs were injected intravenously immediately post implantation. Harvested graft implants were assessed histologically and immunohistochemically. We found that acute rejections were milder in the Decellularized Xenograft and Decellularized Xenograft+ADMSC groups than in the Xenograft group. Mild inflammatory cell infiltration and reduced collagen deposition were observed in the Decellularized Xenograft+ADMSC group. Additionally, ADMSC administration decreased CD8+ lymphocyte counts but increased CD163+ cell counts. In the Decellularized Xenograft+ADMSC group, serum levels of vascular endothelial growth factor and IL-10 were elevated and tissue deposition of IgM and IgG was low. The significant immunosuppressive effects of ADMSCs illustrate their potential use as immunosuppressive agents for xenogeneic biomaterial-based implants.

## Introduction

Various biomaterials have been used for reconstructive surgeries. Decellularized organs or tissues are used to produce extracellular matrix (ECM) scaffold-based biomaterials, which are clinically applied for orthopedic applications and the reconstruction of vessels, heart valves, urinary tracts, skin, and dura mater.^1^ These biomaterials are often crosslinked using bifunctional crosslinking agents, such as glutaraldehyde, to induce tissue sterilization, increase resistance to enzymatic degradation, and reduce tissue antigenicity.^2^ Consequently, the implanted ECM scaffolds cannot assimilate into the surrounding tissues or grow within the host.

Recently, the use of decellularized scaffolds without chemical crosslinking has attracted attention.^3^ Non-crosslinked decellularized scaffolds contain intact native ECM and growth factors,^4^ and can, therefore, be recellularized by host or recipient native cells; the recellularized tissue or organ formed can be orthotopically transplanted for tissue or organ replacement therapy. In animal-based pilot studies, this strategy has been employed for engineering and reconstructing several tissues and organs, including the heart, liver, lung, kidney, esophagus, and small intestine.^5–7^

The ECM scaffolds of mammals contain several proteins, such as collagen, laminin, proteoglycan, and elastin.^8^ Although ECM scaffolds of both human (Alloderm) and animal (Hancock II for porcine and PERIMOUNT Magna for bovine) origin are commercially available, porcine decellularized ECM scaffold without chemical crosslinking represents the most promising ECM scaffold biomaterial owing to its tissue compatibility and the amount of organ and tissues available.^9^ Generally, non-crosslinked decellularized biomaterials induce mild immunoreactions,^10^ which are attributed to the presence of immune-reactive glycolipids in the ECM.^11^

Moreover, decellularized scaffolds might be recognized as foreign substances by immunocompetent cells. Therefore, specific immunosuppression strategies are necessary for engineered organ and tissue engraftment in living organisms.

Mesenchymal stem/stromal cells (MSCs) are multi-potential progenitor cells capable of differentiating into different cell types, such as adipocytes, chondrocytes, and osteocytes. MSCs induce anti-inflammatory and immunomodulatory effects by secreting soluble immunoregulatory molecules, including vascular endothelial growth factor (VEGF), interleukin (IL)-10, transforming growth factor-β (TGF-β),^12^ IL-13,^13^ and hepatocyte-growth factor.^14,15^ Induction of the expression of these factors promotes polarization of macrophages toward the M2 phenotype and inhibition of the growth and function of T-cells, B-cells, and natural killer cells.^16^ Additionally, MSCs inhibit the production of IgM, IgG, and IgA by plasmocytes.^17^ The immunosuppressive potential of MSCs in autoimmune diseases,^18–22^ severe viral pneumonia,^23^ and organ transplantation has been evaluated in clinical trials.^16^ Most *in vivo* studies on MSCs have used bone marrow-derived mesenchymal stem/stromal cells (BMMSCs), since they were the first to be used histologically and their characteristics have been well studied. A previous report showed that mouse BMMSC-seeded cardiac ECM scaffolds exhibited lower inflammation than decellularized scaffolds in a subcutaneous implantation model.^24^ However, adipose-derived mesenchymal stem/stromal cells (ADMSCs) might offer several advantages, such as safety during harvest and efficacious immunosuppression.^25–31^

Human decellularized trachea has been applied to human tracheal transplantation in clinical studies.^32^ This trachea was recellularized by the recipient’s cells. However, this method is costly and time-consuming. Since various nonviable tissues have been used clinically,^33^ decellularized trachea without recellularization may be available for transplantation if the immune response is controlled. If the process of recellularization is omitted, the cost and time would be greatly reduced. Furthermore, the use of xenogeneic biomaterials would be superior in terms of cost and availability, with less ethical restrictions.

In the present study, we used ADMSCs to address the problem of decellularized scaffolds, which induce immunoreactions in living organisms. Accordingly, we evaluated the multi-regulatory immunosuppressive effects of ADMSCs in a xenogeneic decellularized scaffold implantation model, in which pig decellularized bronchi were implanted into rat tracheal defects. We selected pig bronchus since pig-derived biomaterials are the most commonly used xenogeneic material in the clinical settings.

## Results

### Characterization of ADMSCs and ADMSC tropism after venous administration

**Figure 1.**
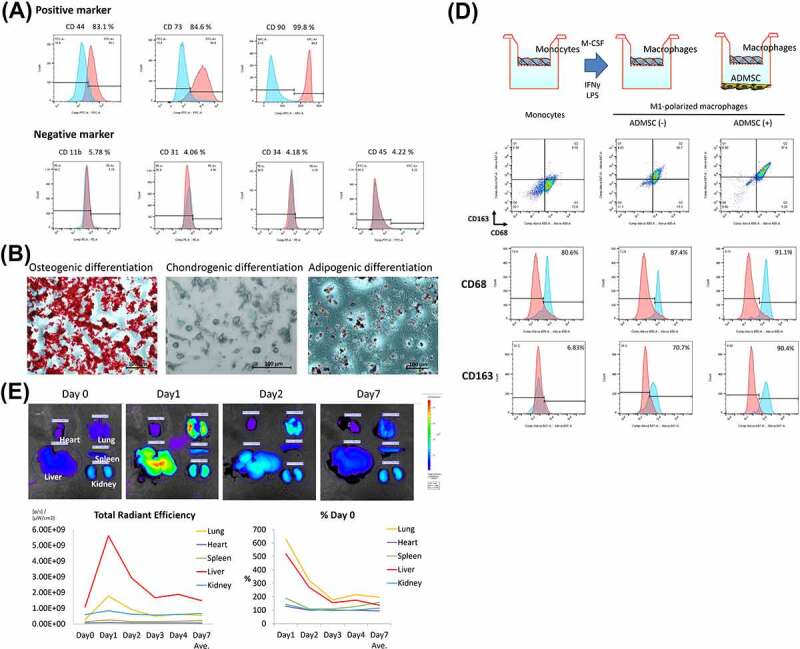
(a) cultured ADMSCs strongly expressed the positive markers CD73, and CD90, in contrast to isotype controls. Expression of the ADMSC negative cell surface markers CD11b, CD31, CD34, and CD45 did not differ from that of the isotype control. (b) adipose-derived mesenchymal stem cells display multilineage differentiation, suggesting that they are ADMSCs. Alizarin red S, alcian blue, and oil red O staining confirmed osteogenic differentiation, chondrogenic differentiation, and adipogenic differentiation, respectively. (c) timeline of the distribution of QD-labeled ADMSCs following intravenous administration. (d) schematic illustration of the experiment design of M2-macrophages polarization by ADMSC co-culture; note that ADMSC co-culture increased the percentage of CD68 (pan-macrophage marker) and CD163 (M2-macrophage marker) and significantly increased CD163 from M1-macrophages.

First, we evaluated the characteristics of ADMSCs harvested from Brown Norway (BN) rats. All cells from the abdominal adipose tissue exhibited a typical spindle-shaped morphology and were positive for ADMSC markers, including CD44, CD73, and CD90, but negative for CD11b/c, CD31, CD34, and CD45, as reported previously (Figure 1a).^34^ We confirmed the ability of ADMSCs to differentiate into osteogenic, adipogenic, and chondrogenic lineages (Figure 1b). Based on these results, we confirmed that these cells exhibited the characteristics of ADMSCs. To examine the intravitreal distribution of intravenously administered ADMSCs in organs, quantum dot (QD)-labeled ADMSCs were injected through the right jugular vein. Interestingly, labeled fluorescence was mainly localized in the liver and lung, peaked on day 1, and could be observed up to day 7 post-injection (Figure 1c).

When rat bone marrow-derived mononuclear cells were differentiated into M1-macrophages and co-cultured with ADMSCs, 90.4% of the cells were CD163 positive compared to the 70.7% in the group without co-culture, suggesting that ADMSCs induce M2-macrophage polarization (Figure 1d)

### Histological evaluation of decellularized trachea

**Figure 2.**
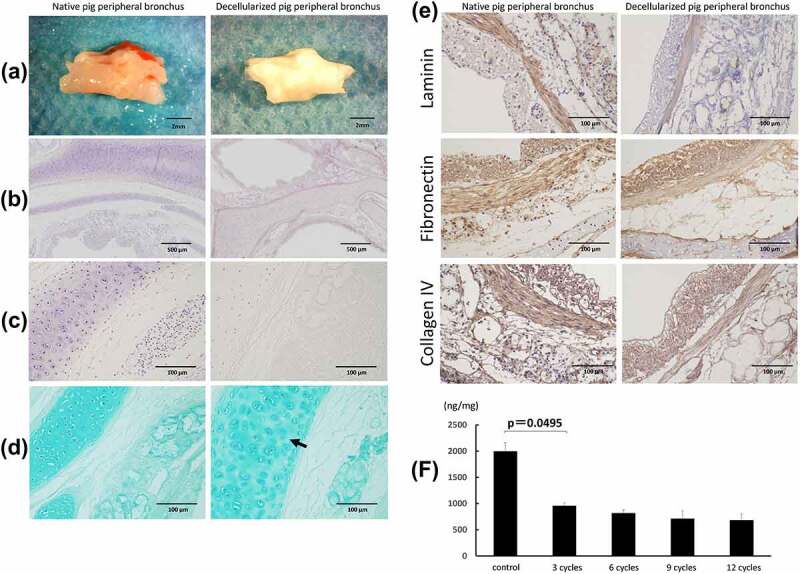
Macroscopic and microscopic findings of a control (non-decellularized) and decellularized bronchus. (a) the decellularized bronchus after nine cycles of the detergent enzymatic method is characterized by complete decolorization and maintains sufficient structural rigidity. (b, c) H&E staining of the decellularized bronchus shows that all cells were removed in the mucosa, submucosa, muscles, and adventitia. Only a small number of nuclei remain in the thick cartilage as compared with that in the native bronchus. (d) alcian blue staining for proteoglycans shows a mild decrease of the cartilage substrate in decellularized bronchial cartilage (arrow). (e) immunohistochemistry for laminin, fibronectin, and collagen IV in the decellularized bronchus. (f) quantification of residual DNA after decellularization of pig bronchus until 12 cycles of detergent enzymatic treatment; *n* = 3 for all groups.

Figure 2a shows the macroscopic images of fresh and decellularized peripheral bronchus of pigs. The decellularized bronchus appeared white in color and maintained sufficient structural rigidity. Hematoxylin staining of the decellularized bronchus showed that all cells were removed in the pericartilage tissues, including the mucosa, submucosa, muscles, and adventitia. Only a small number of nuclei remained in the thick cartilage compared with that in the native bronchus (Figure 2b,c). Alcian blue staining, showing proteoglycans in the bronchial cartilage, indicated a mild decrease of cartilage substrate in the decellularized bronchus (Figure 2d). Immunohistochemical analysis for extracellular matrix proteins including laminin, fibronectin, and collagen IV showed no obvious difference between control and decellularized bronchus, indicating minimal ECM damage in the decellularized scaffold (Figure 2e). The DNA concentration significantly decreased after three cycles of detergent enzymatic treatment, following which the rate of decrease slowed down (Figure 2f).

### Histopathological evaluation after tracheal implantation

**Figure 3.**
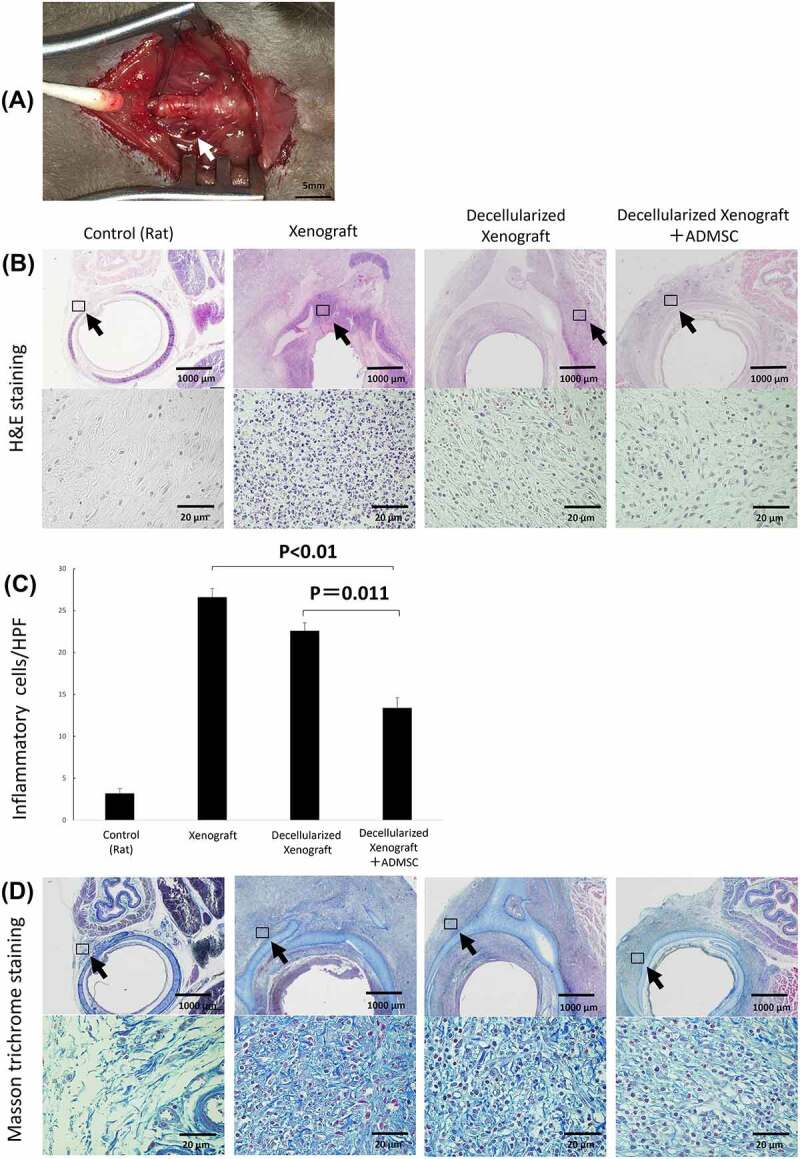
Gross finding of transplanted pig decellularized bronchus at surgery and microscopic findings of implanted pig bronchus in a rat tracheal defect on day 7 post implantation. Xenograft group: BN rats implanted with native pig bronchi; decellularized xenograft group: BN rats implanted with decellularized pig bronchi; decellularized xenograft +ADMSC group: BN rats implanted with decellularized pig bronchi and injected with 1 × 10^6^ ADMSCs. A cross-section of the trachea of a healthy rat is presented for reference. (a) decellularized pig bronchus is sutured at the upper portion of the rat tracheal defect (white arrow). (b) H&E staining shows typical histopathological changes associated with acute rejection, including the destruction of the cartilage matrix and lymphocyte infiltration in the xenograft group. Under high-magnification field views, acute rejection was mild in the decellularized xenograft and decellularized xenograft +ADMSC groups. In particular, the decellularized xenograft +ADMSC group shows low infiltration of inflammatory cells (arrows) and less collagen deposition. (c) inflammatory cell count in each group per high-power field (HPF). Note that the count of inflammatory cells was the lowest in the decellularized xenograft +ADMSC group after the transplantation. (d) Masson’s trichrome staining shows dense fibrotic tissue around the implanted cartilage.

The native porcine bronchus or the decellularized porcine bronchus was transplanted into the BN rat tracheal defect after tracheal resection (Figure 3a). The BN rats were divided into three groups depending on the xenograft used – native pig bronchi (Xenograft group), decellularized pig bronchi (Decellularized Xenograft group), and decellularized pig bronchi with intravenous injection of ADMSCs via the cervical vein (Decellularized Xenograft+ADMSC group). All rats survived until the transplanted grafts were harvested. We evaluated the three groups using hematoxylin and eosin (H&E) and Masson’s trichrome staining (Figure 3b–d). The Xenograft group demonstrated typical histopathological changes associated with acute rejection (i.e., destruction of the cartilage matrix, lymphocyte infiltration into the submucosa in H&E staining, and accumulation of dense fibrotic tissue around the implanted cartilage in Masson’s trichrome staining). Acute rejection was milder in the Decellularized Xenograft and Decellularized Xenograft+ADMSC groups than in the Xenograft group. In particular, the Decellularized Xenograft+ADMSC group showed significantly low infiltration of inflammatory cells in the submucosa (Figure 3b,c) and reduced collagen deposition around the cartilage (Figure 3d).

### Evaluation of immunohistochemistry and immunofluorescence

**Figure 4.**
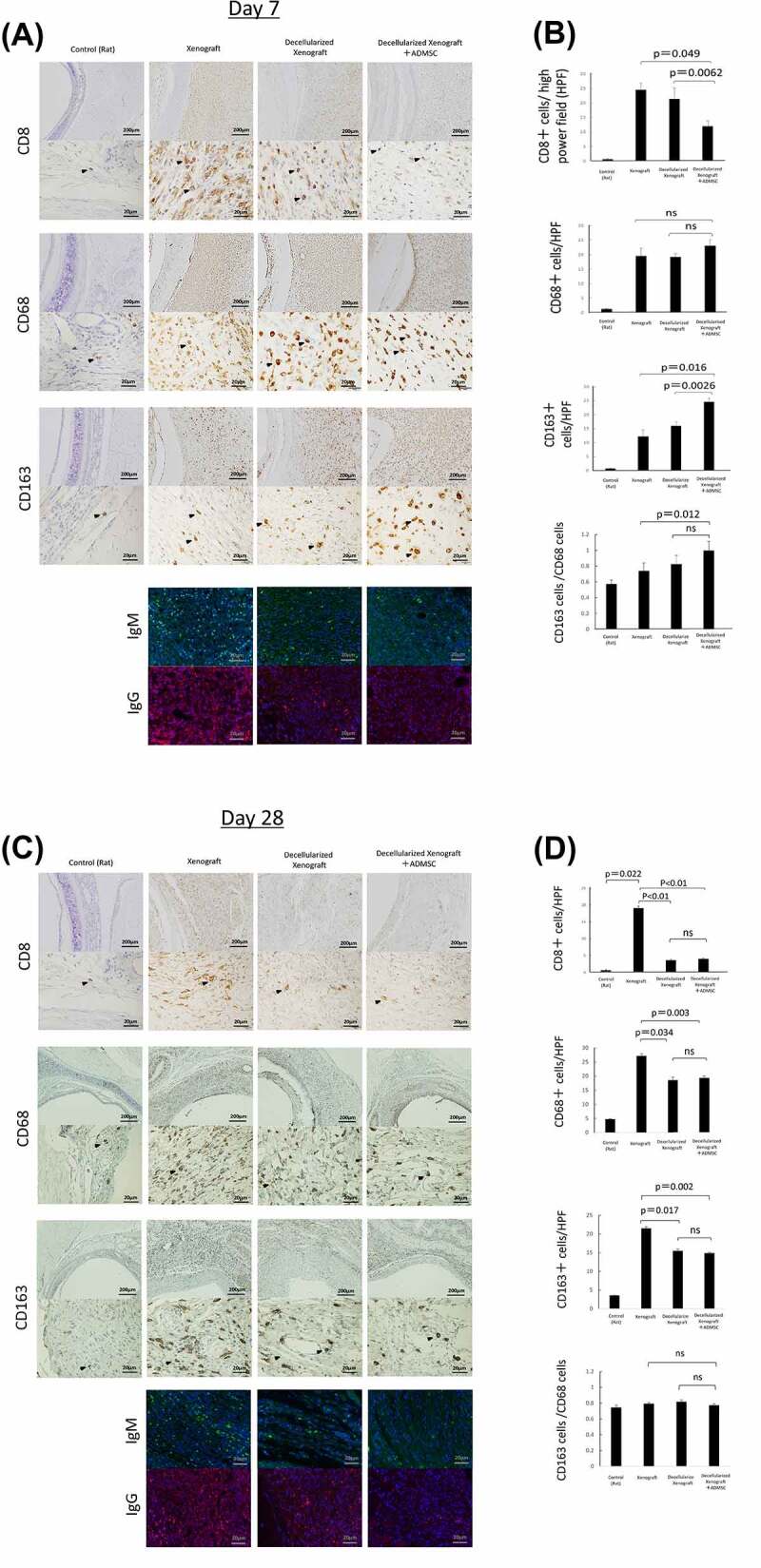
Immunohistochemistry of tissue surrounding the implanted graft on days 0 (*n* = 5), 7 (*n* = 5 or 7), and 28 (*n* = 3) post implantation. (a) low- and high-magnification views of the immunostaining for CD8, a cytotoxic T lymphocyte marker; CD68, a pan-macrophage marker; CD163, an M2 macrophage marker on day 7 post implantation. Arrowheads highlight immunopositive brown cells. The expression of IgM and IgG is reduced in the decellularized xenograft groups and is lowest in the decellularized xenograft +ADMSC group. (b) graphs representing the number of immunopositive cells in five randomly selected high-magnification microscopic fields on day 7 after implantation (*n* = 5 or 7) (400×; 0.0625 mm^2^). (c) low- and high-magnification views of the immunostaining for CD8, a cytotoxic T lymphocyte marker; CD68, a pan-macrophage marker; and CD163, an M2 macrophage marker on day 28 post implantation (*n* = 3). Arrowheads highlight immunopositive brown cells. Expression of CD8, CD68, and CD163 are reduced in the decellularized xenograft and decellularized xenograft +ADMSC groups. The expression of IgM and IgG is reduced in the decellularized xenograft and decellularized xenograft +ADMSC groups. (d) graphs showing the number of immunopositive cells in five randomly selected high-magnification microscopic fields on day 28 post implantation (400×; 0.0625 mm^2^).

Infiltrating macrophages have either a proinflammatory (M1) or anti-inflammatory (M2) phenotype.^35,36^ Immunochemistry results for CD8 (a cytotoxic T lymphocyte marker), CD68 (a pan-macrophage marker), and CD163 (an M2 macrophage marker) in the tissue surrounding the implanted engineered trachea are shown in Figure 4a. We used CD8 expression as a marker for T-cells. The number of CD8+ lymphocytes was significantly lower in the Decellularized Xenograft+ADMSC group relative to the native and Decellularized Xenograft groups (Figure 4b; *p* = 0.049 and *p* = 0.0062, respectively). The number of CD68+ macrophages was similar in all groups. However, comparison with results in the Decellularized Xenograft group revealed that ADMSC administration significantly increased CD163+ cell infiltration into the tissues surrounding the grafts (Figure 4b; *p* = 0.0026). The number of CD163+ cells in the Decellularized Xenograft+ADMSC group was thus significantly higher than that in the native group (Figure 4b; *p* = 0.0016).

Immunofluorescence showed that IgM+ and IgG+ cell counts were lower in the Decellularized Xenograft and Decellularized Xenograft+ADMSC groups as compared with the native group, with scant deposition observed in the Decellularized Xenograft+ADMSC group (Figure 4a). At post-operation day 28, infiltration of CD8+ lymphocytes, CD68+ cells, CD163+ cells, and IgM and IgG deposition were lower in the Decellularized Xenograft group and Decellularized Xenograft+ADMSC group than in the native group. Although the decellularized scaffold showed a very weak immunoreaction compared with the native scaffold, the immunosuppressing effect of ADMSC was not observed at this time point (Figure 4c,d).

### Serum cytokine level analysis

**Figure 5.**
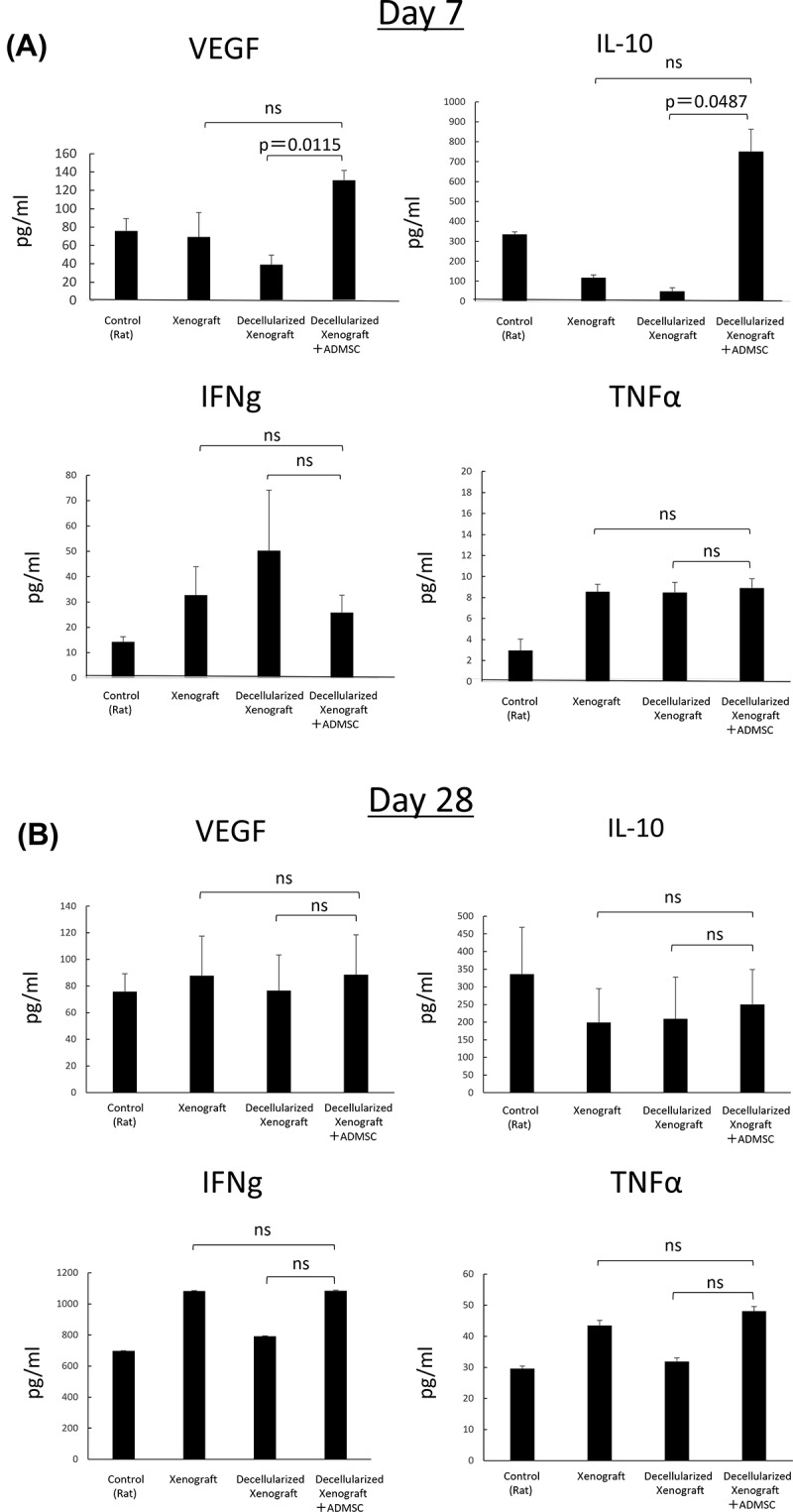
Serum IL-2, IFN-γ, VEGF, IL-10, and TNF-α levels on days 7 (*n* = 5) and 28 (*n* = 3) after implantation. (a) on day 7, serum VEGF and IL-10 levels in the decellularized xenograft+ADMSC group were significantly higher than those in the decellularized xenograft and xenograft groups. Levels of the proinflammatory cytokines IFN-γ and TNF-α are not different between groups. (b) on day 28, no statistical difference was observed among groups.

Serum cytokine levels on day 7 are shown in Figure 5a. Serum VEGF and IL-10 levels in the Decellularized Xenograft+ADMSC group were significantly higher than those in the Decellularized Xenograft group (*p* = 0.0115 and *p* = 0.0487, respectively). Additionally, we observed no differences in serum interferon (IFN)-γ and tumor necrosis factor (TNF)-α levels between groups, and on day 28, serum cytokine levels did not differ between the groups (Figure 5b; *n* = 3).

## Discussion

Decellularized scaffolds without chemical crosslinking are promising biomaterials for tissue reconstruction and engineering.^5,7^ Their use was first applied clinically to tracheal transplantation in 2008.^32^ Decellularized scaffolds are not living tissues, and adaptation of these scaffolds can be achieved by the concomitant infiltration of host cells and escape of immune system cells (mainly phagocytosis by macrophages, antigen presentation by dendritic cells, and antibody production by plasma cells). However, residual growth factors in the decellularized scaffolds can affect cell growth and differentiation,^37,38^ suggesting minimal immunoreactivity. Therefore, the present study determined whether ADMSCs suppress immunoreactions following the implantation of xenogeneic decellularized scaffolds in a rat model of tracheal defect reconstruction. A major finding of the present study was that ADMSCs demonstrated a similar *in vivo* immunosuppressive effect as that observed for BMMSCs, and their administration reduced the immunoreaction to implanted decellularized xenogeneic biomaterials *in vivo*. Accordingly, inflammatory cell infiltration into the surrounding tissue was significantly lower in the decellularized scaffold group than in the native pig bronchus group, with these effects enhanced by the administration of ADMSCs on day 7 post implantation. Masson’s trichrome staining showed mild submucosal fibrosis in the ADMSCs administered group.

MSCs can suppress the entire immune system by exerting multi-regulatory immune-suppressive effects. The underlying mechanisms have been mainly analyzed using *in vitro* and *in vivo* models with BMMSCs. Based on the identity of the antigen-presenting cells, BMMSCs can simultaneously increase macrophage proliferation and migration and induce a pro-tolerogenic shift in polarization from the inflammatory M1 phenotype to the anti-inflammatory M2 phenotype. MSC-reprogrammed M2 macrophages inhibit effector T-cell responses and promote regulatory T-cell proliferation, thereby conferring protection against allograft rejection.^16^ For lymphatic activation, BMMSCs inhibit T-cell proliferation in response to alloantigens and mitogens, prevent the development of cytotoxic T-cells, and induce the production of functional regulatory T-cells.^16^ Moreover, BMMSCs can modulate B-cell functions and inhibit B-cell proliferation and differentiation *in vitro*. ^17,19^

**Figure 6.**
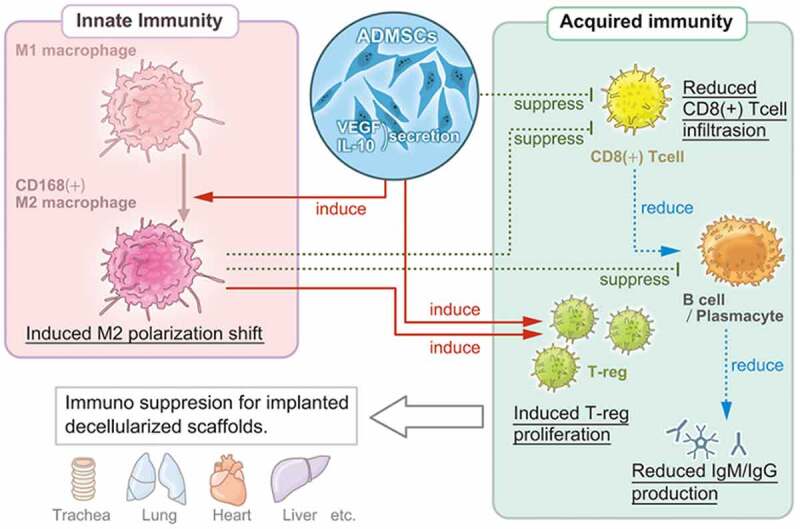
Multi-regulatory immunosuppressive effects of ADMSCs with respect to the implanted decellularized scaffolds. ADMSCs induce a shift in macrophage polarization toward the M2 phenotype and suppress T-cell and B-cell proliferation. The suppression of innate and acquired immunity reduces host immunoreactivity toward decellularized scaffolds.

These processes are induced by the overexpression of several secreted soluble mediators, including indoleamine-pyrrole 2,3-dioxygenase, TGF-β, IL-10, and heme oxygenase-1.^16^ A similar mechanism was demonstrated in the present study. In this model, ADMSC administration significantly increased the serum levels of VEGF and immunosuppressive IL-10. The secretome of ADMSCs contains high levels of VEGF that accelerate wound healing *in vivo*.^39^ The anti-inflammatory effects of IL-10 have been proposed as a major mechanism for suppressing excessive immune responses. IL-10 works on immune cells such as T-cells and macrophages, directly suppressing their activation and weakening their ability to present antigens, thereby curbing the immune response.^40^ Moreover, CD163+ M2 macrophage counts increased in the Decellularized Xenograft+ADMSC group, while ADMSCs suppressed CD8+ cytotoxic T-cell infiltration as well as IgM and IgG depositions. These results indicate that ADMSCs, similar to BMMSCs, exert immunosuppressive effects on macrophage polarization, as well as on T-cell and B-cell activities (Figure 6). However, the immunosuppressive effect of ADMSCs might be broader and stronger than that of BMMSCs, as xenoantibody production by B-cells was lower when induced using BMMSCs than with ADMSCs.^29^ Given the present results and previous findings, an ADMSC-based strategy might offer a promising option in immunosuppressive regimens for biomaterial implantation and the development of strategies for engineered organ and tissue transplantation, especially when using decellularized scaffolds.

In the present study, we injected ADMSCs intravenously. The route/number was selected based on our previous study, which showed that ADMSCs suppress immune responses in a lung transplantation model. We did not inject the ADMSCs directly into the grafted area as it seemed impossible to inject ADMSCs evenly over the circumference of the graft. Heterogeneity in the localization of ADMSCs might affect the results.

QD-tracked ADMSC-based studies can provide insights into the tropism and persistence of intravenously injected ADMSCs. The present study showed that intravenously infused ADMSCs first distributed to the lungs and the liver, peaked on day 1, and started to fade thereafter until day 7, which agreed with the findings of a previous study involving BMMSCs.^41^ Furthermore, in a study in which human MSCs were injected in mice, human DNA in the lungs, which was detected after 24 h, was no longer detectable on day 7, with no trace in other organs.^42^ Accordingly, the present study indicated that the immunosuppressive effect of ADMSCs might have disappeared by day 28. Notably, cytokine levels, including those of VEGF and IL-10, were nearly the same between groups on day 28 post implantation, and CD8+ cytotoxic T-cell infiltration was the same in the Decellularized Xenograft and Decellularized Xenograft+ADMSC groups. Interestingly, CD8+ cytotoxic T-cell infiltration remained high in the Xenograft group, suggesting that decellularized scaffolds themselves have very weak immunoreactivity compared with the native xenogeneic bronchus. Interestingly, CD8+ cytotoxic T-cell infiltration remained high in the Xenograft group, implying that decellularized scaffolds have very weak immunoreactivity compared with the native xenogeneic bronchus.

Moreover, histologic examination of the decellularized pig bronchus showed that our decellularization protocol could not remove chondrocytes, which contain DNA, from the scaffold, since cartilage is a dense tissue, making penetration of chemical agents^43,44^

The residual DNA content of the decellularized pig bronchial scaffold was 10 times higher than the reported criteria^45^ even after 12 cycles of detergent enzymatic treatment.^46,47^ However, severe decellularization is a “double-edged sword,” as it allows for minimal DNA residue but in exchange for substantial matrix damage, including loss of GAGs, elastin, fibronectin, and laminin.^48^ Accordingly, severely decellularized tissues elicited a more exacerbated inflammation than mild decellularization despite containing a large amount of DNA.^46^ One study on commercially available ECM scaffold materials showed that although remanent DNA fragments are common, they do not present a significant risk of rejection, suggesting that the presence of some remanent nuclear material is acceptable.^49^ Porcine-derived ECM of the small intestinal submucosa implanted in BALB/c mice induces a T-helper cell 2-type response and leads to implanted graft acceptance.^50^ Reports on the use of decellularized tissue scaffolds in clinical practice are limited, but transplanted recellularized trachea, which harbors remanent donor cellular elements in cartilaginous areas, is protected from rejection without the use of immunosuppressive drugs.^32^ Since matrix damage is more important than DNA persistence, the present study allowed for a large amount of DNA to remain in decellularized porcine bronchi. Decellularization changes the mechanical properties of the decellularized scaffold,^51^ which might affect the stiffness of the scaffold and induce stenosis. Therefore, we used a stent to prevent stenosis of transplanted decellularized pig bronchi. Intra-tracheal stents will be necessary until the transplanted decellularized trachea regain strength through *in vivo* reconstruction.

This study has several limitations. First, we did not measure the residual sodium deoxycholate in the decellularized biomaterials, as it is challenging. Furthermore, injection of ADMSCs along with the native implant was not evaluated. A previous study of BMMSC administration in a murine model of heterotopic tracheal transplantation showed that BMMSCs inhibited intraluminal obstruction of the tracheal allograft, reduced collagen deposition, and decreased TGF-β expression to similar levels as observed in the isograft-implanted model.^52^ Therefore, although we did not experimentally confirm this in the present study, based on available evidence, we presume that similar attenuation of inflammation would be observed in native implants with ADMSC administration.

In summary, intravenously administered ADMSCs exhibited significant immunosuppressive effects on xenogeneic decellularized ECM-based biomaterial in a rat model at least 7 days post implantation. These effects were similar to the multi-immunoregulatory effects of BMMSCs, which induce immunosuppressive M2 macrophage infiltration and reduce cytotoxic T-cell invasion and immunoglobulin deposition via cytokine secretion by ADMSCs. This immunosuppressive effect of ADMSCs suggests that their administration is beneficial for biomaterial implantation and future reconstructive medicine, including engineered tissue and organ transplantation.

## Materials and methods

### ADMSC preparation

ADMSCs were prepared, as previously described.^31^ Briefly, abdominal adipose tissue was surgically excised from 5- to 7-week-old male BN rats (Charles River Laboratories). Only male rats were used to avoid possible hormonal influences of menstruation on the viability of MSCs, including cytokine secretion capacity, in female rats.^53^ Adipose tissue was digested with 0.001% collagenase (Celease; Cytori) at 37°C for 30 min. After several cycles of shaking and centrifugation, the sample preparation was filtered through a 100-μm pore nylon mesh (Corning). Mesenchymal cells were separated via centrifugation and then resuspended in OriCellTM mesenchymal stem cell growth medium (DS Pharma Biomedical).

The cells were cultured at 37°C in 100-mm dishes for 14 to 18 days until reaching confluence. Aliquots of 1 × 10^6^ primary cells and passaged cells (through three passages) were used in subsequent *in vivo* and *in vitro* experiments after reaching confluence.

### Phenotypic characterization and multilineage differentiation of ADMSCs

Phenotypic characteristics of the stem cells were evaluated via flow cytometric analysis, as previously reported.^31^ Briefly, stem cells derived from adipose tissue were labeled for 20–30 min on ice with fluorescence-molecule-conjugated antibodies to detect the positive markers CD44 (BD Biosciences, 550974), CD73 (BD Biosciences, 551123), and CD90 (BD Biosciences, 554897), as well as the negative markers CD11b (BD Biosciences, 561691), CD31 (BD Biosciences, 555027), CD34 (Santa Cruz Biotechnology, sc-7324), and CD45 (BD Biosciences, 561867) or the isotype control. Primary cells that had reached confluence were used for a multilineage differentiation assay. Differentiation was induced by culture for 14–28 days in either mesenchymal stem cell osteogenic, chondrogenic, or adipogenic differentiation medium with the appropriate supplements (DS Pharma Biomedical). Differentiation into each lineage was confirmed using the following stains: Alizarin Red S for osteogenic differentiation, Alcian Blue for chondrogenic differentiation, and Oil Red O for adipogenic differentiation.

### Tropism and persistence of intravenously administered ADMSCs traced using QDs

The inorganic probes (QDs) comprised CdSe/ZnScore/shell semiconductor nanocrystals.^54,55^ The transduction of QDs with octa-arginine peptide (R8) has previously been used to label ADMSCs and maintain stem cell potency with low cytotoxicity, as previously described.^54,55^ Cultured ADMSCs were incubated with the R8–QD complex (2 nM), and QD-labeled ADMSCs (1 × 10^6^ were injected intravenously into 8- to 11-week-old male rats. Rats were anesthetized and maintained under anesthesia using isoflurane (2–4%; 3‒5 L/min), and were later sacrificed. Fluorescence levels of the harvested organs were tracked and measured using the IVIS Lumina Series III system (PerkinElmer) until 7 days post ADMSC injection.

### ADMSC/Macrophage co-culture

Influence of ADMSCs on monocyte to macrophage polarization or activation to M1 and M2 functional states were evaluated by culturing both the cell types together in 12 well culture plates (Falcon, Corning) physically separated using cell-culture inserts (0.4 microns, Millipore). BN rats (8- to 12-week-old) were sacrificed via carbon dioxide (CO_2_) inhalation, and the femur and tibia were collected aseptically. Bone marrow was flushed out using RPMI 1640 culture medium and collected by centrifugation (5 min, 5,000* × g*), cells were separated with a 40 μm cell strainer. Thereafter, the cells were cultured in RPMI 1640 supplemented with 10% FBS, 100 U/mL penicillin, 0.1 mg/mL streptomycin, 2 mM L-glutamine, and 50 ng/mL recombinant rat macrophage colony-stimulating factor (M-CSF, Pepro Tech). After 3 days, cells differentiated into naïve macrophages. Naïve macrophages were further polarized to inflammatory M1 state by culturing them for 30 h in complete media containing 15 ng/mL IFNγ (Invitrogen) and 100 ng/mL lipopolysaccharide (Sigma). For macrophage polarization, 3.5 × 10^5^ M1 macrophages were seeded on culture inserts and co-cultured with 1.5 × 10^4^ ADMSCs (plated in the bottom chamber) for 18 h. Macrophages were harvested for flow cytometry assessment of specific cell surface antigens. CD11b (Biolegend; 1:200; 201807) positive cells were further separated based on CD68 (Abcam; 1:200; ab31630) and CD163 (Abcam; 1:200; ab182422) expression.

### Animals

Peripheral bronchi were harvested from 3-month-old male three-way cross pigs (Landrace, Large White, and Duroc) weighing 16 kg. Pig bronchi were implanted in 8- to 12-week-old BN rats (Charles River Laboratories), which were housed separately and maintained in a barrier facility with temperature control (22–24°C) and an automatic 12-h light/dark cycle (lights on at 5:00 A.M.). All rats received food *ad libitum* (Charles River Formula-1; Oriental Yeast Co.). The study design was approved by the institutional ethics committee (protocol ID: 140811-1-4). All animal studies were performed according to the guidelines of the institutional animal care and ethics committee of Nagasaki University.

### Experimental design

Experimental animals were divided into three groups: BN rats implanted with native pig bronchi (Xenograft group; *n* = 8), BN rats implanted with decellularized pig bronchi (Decellularized Xenograft group; *n* = 8), or BN rats implanted with decellularized pig bronchi and intravenously injected with 1 × 10^6^ ADMSCs via the cervical vein (Decellularized Xenograft+ADMSC group; *n* = 10). Immunosuppressive agents were not used in any group. The implanted grafts were harvested on days 7 (*n* = 5 for Xenograft group and Decellularized Xenograft group, *n* = 7 for Decellularized Xenograft+ADMSC group) and 28 (*n* = 3 for each group) for histologic and immunohistochemical analyses.

### Bronchial graft decellularization

Pigs were sedated using intramuscular injections of ketamine (10 mg/kg) and midazolam (2 mg/kg). After sedation, 2% isoflurane was supplied to the standard respiratory system to sustain anesthesia. During anesthesia, the lungs were excised, and bronchi (1‒2 cm in length) were harvested. All connective tissues were excised, and the bronchi were rinsed with phosphate-buffered saline (PBS, pH 7.4; Wako Pure Chemical Co.). Bronchial segments, 5-mm-long with a 4-mm diameter, were taken at the fourth or fifth order branch of the pig bronchus. The internal diameter of the pig bronchus was the same as that of the rat trachea. Bronchial segments were divided into two groups: a native pig bronchial group (Xenograft) and a Decellularized Xenograft group. Bronchi in the native group were stored at 4°C in PBS supplemented with 1% penicillin, streptomycin, and amphotericin B (Wako Pure Chemical Co.). The bronchi in the other group were decellularized using nine cycles of detergent enzymatic treatment, as described previously.^32^ Bronchial segments were shaken in 4% sodium deoxycholate (Sigma-Aldrich) at 37°C for 4 h, sodium chloride (1 mM) containing 5,000 U/mL DNase (Sigma-Aldrich) at 37°C for 3 h, and stored at 4°C in PBS supplemented with 1% penicillin, streptomycin, and amphotericin B.

### Evaluation of DNA content in the pig bronchi

The DNA in the pig bronchi was purified using the QIAamp DNA Mini Kit (QIAGEN, Hilden, Germany) following the manufacturer’s protocol. Briefly, 25 mg decellularized pig bronchi was thoroughly minced into small pieces. After adding 20 µL Proteinase K, the sample was incubated at 56°C until the tissue was completely lysed. The solution was then centrifuged, and the supernatant was collected. Ethanol (200 µL; 96–100%) was added to the supernatant, and the mixture was pulse vortexed for 15 s, after which it was spun down for a few seconds, and the supernatant was collected. The DNA content in each sample was measured in triplicate using a Nanodrop Spectrophotometer (ND-1000) V3.7 (NanoDrop Technologies, Inc. DE, USA) following the manufacturer’s protocol.

### Transplantation of the pig bronchus and ADMSC administration

Eight- to 11-week-old male rats were used as recipients. Rats were anesthetized and maintained using isoflurane (2–4%; 3‒5 L/min). A midline cervical incision was performed to expose the entire trachea, and three rings of tracheal segments below the cricoid cartilage were resected and replaced with the decellularized pig bronchi supported by a silicone stent tube (1.5 mm internal diameter; Kenis). Proximal and distal end-to-end anastomoses were made with 7–0 Prolene sutures (Ethicon), and the cervical incision was closed in layers with 4–0 Vicryl sutures (Ethicon). After intravenous injection of heparin (1.000 U/kg) into the left jugular vein, recipients of the Decellularized Xenograft+ADMSC group were injected with heparinized 1 × 10^6^ ADMSCs dissolved in 1 mL PBS via the same route until 30 min post implantation. The administered ADMSC amount was decided based on a previously published study on ADMSC-induced immunosuppressive lung transplantation.^31^

### Histopathology studies

Grafts were harvested from euthanized recipient rats on days 7 and 28 post implantation. Native and decellularized trachea samples were fixed in 10% buffered formalin at room temperature (20–25°C) for 24 h. The specimens were embedded in paraffin, sectioned into 4-µm-thick slices, and stained with H&E and Masson’s trichrome. Proteoglycans were visualized using Alcian blue staining. The middle portion of the implanted grafts and the area around the tissue were assessed.

### Immunohistochemistry and immunofluorescence

Samples were collected on days 7 and 28 post implantation, fixed in formalin, and embedded in paraffin blocks as described above. The 4-µm-thick formalin-fixed, paraffin-embedded tracheal sections were used for immunochemistry and immunofluorescence. The middle portion of the implanted grafts and the area around the tissue were assessed. Antigen retrieval was performed in 10 mM citrate buffer (pH 6) at 121°C for 15 min. Slides were incubated in 3% H_2_O_2_ for 30 min at room temperature (RT) for endogenous peroxidase inactivation. Blocking was performed with 5% normal goat serum in PBS for 1 h. Slides were incubated with primary antibodies overnight at 4°C, followed by secondary antibodies for 1 h at RT. Then the slides were mounted with 3′-3′-diaminobenzidine (DAB). The following primary antibodies were used: rabbit anti-CD8 (Abcam; 1:200; ab33786), mouse anti-CD68 (Abcam; 1:500; ab31630), rabbit anti-CD163 (Abcam; 1:500; ab182422), anti-laminin (Abcam; 1:100; ab11575), anti-fibronectin (Abcam; 1:100; ab6328), and collagen IV (Abcam; 1:50; ab6586). Peroxidase-conjugated IgG polyclonal antibody was used as the secondary antibody (Histofine simple stain MAX-PO, Nichirei, Japan). The absolute number of CD8+, CD68+, and CD163+ cells was counted in five randomly selected high-power microscopic fields (400×; 0.0625 mm^2^), and the average counts were recorded.

For immunofluorescence assays, tissue sections were fixed in 4% paraformaldehyde, blocked with 1% bovine serum albumin prepared in PBS containing 0.3% Triton X-100 for 1 h, and probed overnight with primary antibodies against rabbit anti-rat IgG (Abcam; 1:200; ab6734) and goat anti-rat IgM (Abcam; 1:200; ab97178). Secondary antibodies were goat anti-rabbit IgG H&L (Alexa Fluor® 594) preadsorbed (ab150084) for IgG and donkey anti-goat IgG H&L (Alexa Fluor® 488) (ab150129) for IgM.

All stained sections were mounted in mounting medium containing 4,’6-diamidino-2-phenylindole (DAPI; Vector Laboratories, CA) and imaged using a Leica DM6000 (Leica) or BZ-9000 BioRevo (Keyence) microscope.

### Serum cytokine level measurements

For the cytokine assays, 7 mL of whole blood was collected via the inferior vena cava and placed into EDTA tubes (NIPURO; NP-EK0205 30,305) on day 7 post implantation and centrifuged at 3,000 rpm (1,200 ×*g*) to obtain serum. We quantitatively determined the steady-state level of the circulating proinflammatory and anti-inflammatory cytokines IL-2, IFN-γ, VEGF, IL-10, and TNF-α using the MILLIPLEX MultiAnalyte profiling rat cytokine/chemokine panel (Millipore) run on a Luminex platform (Luminex Corp.). For quality assurance, each sample was run twice, and the mean was used as the index value for each sample. Additionally, two kit-supplied quality controls were run on each plate in duplicate and confirmed to fall within the expected range. Whole plates were read using a multiplex plate reader and companion software. All cytokine and chemokine concentrations are reported in pg/mL.

### Statistical analysis

For comparison of values among groups, Kruskal – Wallis tests were used to determine differences between median values, with a Steel – Dwass test used when the difference was significant. The Mann – Whitney *U* test was used to analyze enzyme-linked immunosorbent assay results. A *P* < 0.05 was considered significant. Analyses were performed using JMP software (v.10.0.2; SAS Institute, Cary, NC, USA).

## Data Availability

Data that support the findings of this study are available from the corresponding author upon reasonable request.
